# Detection of Lard in Cocoa Butter—Its Fatty Acid Composition, Triacylglycerol Profiles, and Thermal Characteristics

**DOI:** 10.3390/foods6110098

**Published:** 2017-11-09

**Authors:** Marliana Azir, Sahar Abbasiliasi, Tengku Azmi Tengku Ibrahim, Yanty Noorzianna Abdul Manaf, Awis Qurni Sazili, Shuhaimi Mustafa

**Affiliations:** 1 Halal Products Research Institute, Universiti Putra Malaysia, 43400 UPM Serdang, Selangor, Malaysia; marlianaazir@gmail.com (M.A.); yanty@upm.edu.my (Y.N.A.M.); awis@upm.edu.my (A.Q.S.); 2 Department of Microbiology, Faculty of Biotechnology and Biomolecular Sciences, Universiti Putra Malaysia, 43400 UPM Serdang, Selangor, Malaysia; 3 Bioprocessing and Biomanufacturing Research Center, Universiti Putra Malaysia, 43400 UPM Serdang, Selangor, Malaysia; 4 Institute of Bioscience, Universiti Putra Malaysia, 43300 Serdang, Selangor, Malaysia; tengkuazmi@upm.edu.my; 5 Faculty of Veterinary Medicine, Universiti Putra Malaysia, 43400 UPM Serdang, Selangor, Malaysia; 6 Department of Animal Science, Faculty of Agriculture, Universiti Putra Malaysia, 43400 UPM Serdang, Selangor, Malaysia; 7 Institute of Tropical Agriculture and Food Security, Universiti Putra Malaysia, 43400 UPM Serdang, Selangor, Malaysia

**Keywords:** cocoa butter, lard, fatty acids methyl ester, triacylglycerol, adulteration, food

## Abstract

The present study investigates the detection of lard in cocoa butter through changes in fatty acids composition, triacylglycerols profile, and thermal characteristics. Cocoa butter was mixed with 1% to 30% *(v/v*) of lard and analyzed using a gas chromatography flame ionization detector, high performance liquid chromatography, and differential scanning calorimetry. The results revealed that the mixing of lard in cocoa butter showed an increased amount of oleic acid in the cocoa butter while there was a decrease in the amount of palmitic acid and stearic acids. The amount of POS, SOS, and POP also decreased with the addition of lard. A heating thermogram from the DSC analysis showed that as the concentration of lard increased from 3% to 30%, two minor peaks at −26 °C and 34.5 °C started to appear and a minor peak at 34.5 °C gradually overlapped with the neighbouring major peak. A cooling thermogram of the above adulterated cocoa butter showed a minor peak shift to a lower temperature of −36 °C to −41.5 °C. Values from this study could be used as a basis for the identification of lard from other fats in the food authentication process.

## 1. Introduction

Worldwide, there is an ever increasing demand by consumers for information and confidence pertaining to the origin and content of purchased food. In this respect, food manufacturers have no alternative but to provide and confirm the authenticity of the origin of their food ingredients. This pressing demand, accompanied by legislative and regulatory drives, has increased the complexity and level of regulation imposed on food production. Protecting consumer rights becomes the foremost issue and constitutes important challenges facing the food industry [1].

The replacement of expensive oils by comparatively cheaper ones is a common practice from an economic point of view. There is also a tendency for oil to be substituted in view of its high price, increased demand, limited availability, and accessibility. Cocoa butter is a byproduct of cocoa. As it constitute an expensive component of chocolate and plays an important role in the melting properties of chocolate, its availability in the market is most often unpredictable. Its demand in the food and pharmaceutical industries is very high by virtue of its physical properties and organoleptic qualities. For a long time, there has been considerable effort to replace it either fully or partially with other vegetable fats, the so-called cocoa butter alternatives, which would be much cheaper. Lard could be another alternative for cocoa butter as it is the cheapest form of fat readily available for use by the food industries. Lard or industrially modified lard could be effectively blended with other vegetable oils to produce shortenings, margarines, and other food oils. There is, however, a limitation on the use of this animal product in the food industry from the perspective of the Muslim religion, in addition to the risk of biological complications and health risks associated with daily intake [2].

The ever increasing price of organic products and limited availability brought about by high demand has led to an increased number of fraudulent practices in the food industry which calls for a reexamination of the procedures with respect to their authentication to reassure both consumers’ confidence and fair trade practices. In this context, it is pertinent to develop analytical procedures capable of detecting fraudulent practices and protect the consumers from misleading labeling and unsubstantiated claims.

Using physical properties such as the refractive index, viscosity, melting point, saponification, and iodine value are no longer practical to detect adulteration in view of the availability of more current, sophisticated procedures and approaches. However, each oil and fat has a specific component at a known level and their presence and quantity should be considered as a detection tool. Therefore, advanced and sophisticated methods with high sensitivity to detect and quantify adulteration need to be given due consideration [3].

Several methods have been employed to detect lard in foods and food products which include DNA-based polymerase chain reaction (PCR), Fourier transform infrared spectroscopy (FTIR), electronic nose technology (e-nose), differential scanning calorimetry (DSC), and chromatographic-based techniques (Table 1). However, to date there is no report published on the application of a gas chromatography flame ionization detector (GC-FID), high performance liquid chromatography (HPLC), and DSC for the detection and quantification of lard in cocoa butter. Therefore, this study is aimed at determining the level of lard adulteration in cocoa butter using DSC, HPLC, and GC-FID.

## 2. Materials and Methods

### 2.1. Sample Preparation and Supplies

Pig adipose tissue was obtained from a local market at Sri Serdang, Selangor, Malaysia, while cocoa butter (CB) was kindly donated by the Malaysian Palm Oil Board (MPOB). Acetone (C_3_H_6_O, ≥99.9%), acetonitrile (CH_3_CN, ≥99.0%), chloroform (CHCl_3_, ≥99.8%), anhydrous sodium sulfate (Na_2_SO_4_), cyclohexane (C_6_H_12_), Wij’sreagent (Iodine trichloride solution), potassium iodide (KI), sodium thiosulphate pentahydrate (Na_2_S_2_O_3_·5H_2_O), and acetic acid glacial (CH_3_CO_2_H, ≥99.0%) were supplied from Orec, New Zealand. Triacylglycerol (TAG) standards and the sodium methoxide solution (CH_3_ONa, 25 wt % in methanol) were sourced from Sigma-Aldrich (St. Louis, MO, USA). All chemicals used in this study were of analytical grade.

### 2.2. Extraction of Oil from Lard

The oil extraction procedure is as described by Hoffmann [37]. Briefly, the animal fat samples were diced into pieces measuring 0.5 cm × 0.5 cm, heated at 90–100 °C for 2 h, and strained through a triple folded muslin cloth to remove impurities. The melted fats were filtered through Whatman No. 2 filter paper containing Na_2_SO_4_ flushed with nitrogen to prevent oxidation [38] and stored in a tightly closed container at 4 °C until use. 

### 2.3. Blend Preparation of Adulterated Cocoa Butter

Cocoa butter and lard were melted at 60 °C and blended in proportions of 99:1, 97:3, 95:5, 90:10, 85:15, 80:20, 75:25, and 70:30 (g/100 g).

### 2.4. Determination of Triacylglycerol Composition of Lard, Cocoa Butter, and Their Admixture

The determination of the TAG composition of lard, cocoa butter, and adulterated cocoa butter with 1% to 30% of lard was carried out according to the procedure described by Haryati et al. [39]. Briefly, 0.1 g of the sample was dissolved in 1 mL of chloroform. The composition of TAG was determined by HPLC (Waters Model 510, Waters Associates, Milford, MA, USA). The HPLC system was equipped with a LiChrosphere^®^ RP-18 column (5 μm particle size, 12.5 cm × 34 mm) (Merck, Darmstadt, Germany) and RID detector (Model 410, Waters Associates, Milford, MA, USA). A mixture of acetone: acetonitrile (63.5:36.5) was used as the solvent for elution at a flow rate of 1.5 mL/min. The column temperature was maintained at 30 °C. All measurements were carried out in triplicate. Peaks for the respective samples were identified using a set of TAG standards.

### 2.5. Determination of Fatty Acid Composition of Lard, Cocoa Butter, and Their Admixture

Fatty acids methyl ester (FAME) was prepared according to the method of Marina et al. [40]. A total of 50 mg of each sample was dissolved in 0.8 mL of hexane and 0.2 mL of 1 M sodium methoxide. The mixture was vortexed for 1 min and 1 µL of the clear supernatant was subsequently injected into a gas chromatograph (Shimadzu GC-14 A) equipped with an FID detector (Shimadzu, Vienna, Austria). A polar capillary column BPX70 (0.32 mm internal diameter, 30 m length and 0.25 mm film thickness; SGE International Pty, Ltd., Victoria, Australia) was used at a column pressure of 10 psi. The initial column oven temperature was set at 90 °C, and programmed to increase to 220 °C at 15 °C/min (for 5 min), 2 °C/min (for 20 min), and 15 °C/min (for 1 min). Temperatures of the injector and detector were maintained at 240 °C. Peaks of the respective samples were identified by comparing their retention time with certified reference standards of FAME (Supelco, Bellefonte, PA, USA). The area percent of each fatty acid was calculated by dividing its peak area by the total peak area of the fatty acids identified.

### 2.6. Thermal Analysis of Lard, Cocoa Butter, and Their Admixture Using Differential Scanning Calorimetry

The thermal characteristics of the oils were analyzed using DSC (Mettler Toledo, Greifensee, Switzerland). Four to eight mg of oils was weighed into aluminium pans sealed hermetically and analyzed using a DSC Q100 instrument (TA Instruments, New Castle, DE, USA). Nitrogen (99.99% purity) was purged at a flow of 20 mL/min. The instrument was calibrated using indium (m.p. 156.6 °C, ∆H_f_ = 28.45 J/g) and n-dodecane (m.p. −9.65 °C, ∆H_f_ = 216.73 J/g), and an empty pan was used as reference [41]. All samples were subjected to the following temperature programs where the samples were cooled from 50 °C to −70 °C, held for 5 min, and heated from −70 °C to 50 °C at a rate of 5 °C/min. The melting and crystallisation parameters of each sample were obtained using Mettler Toledo STAR^e^ software system (STAR^e^ SW 9.20 software, Greifensee, Switzerland). Thermograms were analyzed with universal analysis software (Version 3.9A, TA Instruments, New Castle, DE, USA) to obtain the enthalpy (∆H, J/g), T_on_ (°C), and T_off_ (°C) of the transitions (intersection of baseline and tangent at the transition) and peak temperature (Tp °C). The range of the transitions was calculated as the temperature difference between T_on_ and T_off_. All experiments were determined in triplicate and the average of three measurements was used for data analysis.

### 2.7. Statistical Analysis

Data obtained were analyzed using a one-way analysis of variance (ANOVA) test. The Tukey’s honest significant difference test (HSD) method was chosen for the post-hoc tests for each dataset. The Tukey Honestly Significant Difference (HSD) function of SPSS (Version 14.0, SPSS Inc., Chicago, IL, USA) was used to compare the means of all data. All statistics were based on a confidence level of 95%, and *p* < 0.05 was considered statistically significant.

## 3. Results and Discussion

### 3.1. Triacylglycerol Composition of Lard, Cocoa Butter, and Their Admixture

**Figure 1:**
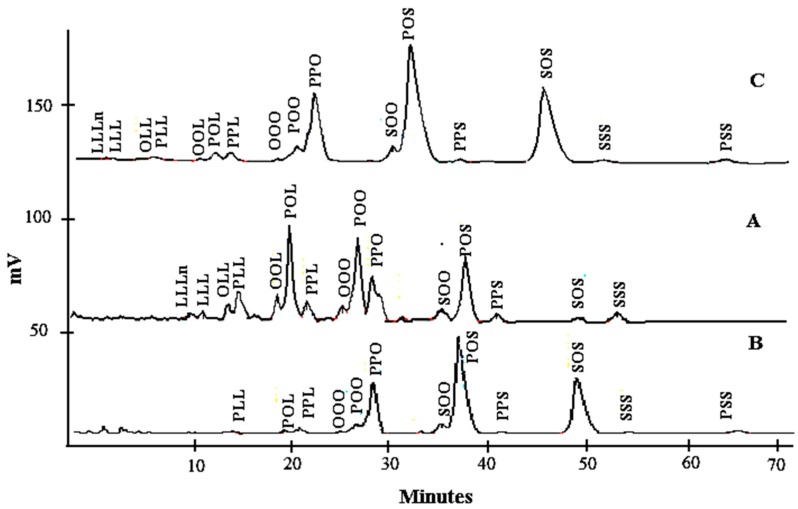
The chromatogram of lard (**A**), cocoa butter (**B**), and their admixture (**C**).

The TAG profiles of lard, cocoa butter, and their admixture are shown in Table 2. The distinction in the nature of TAG is the principal factor that makes fats different from one another and these variations affect the TAG separation. The total di-unsaturated TAGs in LD (48.03%) were higher compared to those of cocoa butter, while cocoa butter had higher total di-saturated TAG values (90.55%). Moreover, a relatively high proportion of L, Ln, and O present in lard was reflected by the shorter retention times of TAGs such as linolenoyldilinoleoylglycerol (LLLn), trilinolein (LLL), dilinoleoyloleoylglycerol (OLL), linoleoyldioleoylglycerol (OOL), dilinoleoylpalmitoylglycerol (PLL), linoleoyloleoylpalmitoylglycerol (POL), and dioleoypalmitoylglycerol (POO) (Figure 1). POL (20.21%), POS (18.58%), and POO (17.25%) in lard and POS (41.67%), SOS (28.47%), and POP (19.13%) in cocoa butter are the most abundant TAG. However, this total amount in cocoa butter is slightly different from that reported by Shukla [42], although in this research, POS (38.5%), SOS (30.30%), and POP (15.20%) were the major TAGs. This variation could be due to the different methods of extraction, ripeness values, cultivar types, growing conditions, and the origin of cocoa.

The levels of TAGs containing S viz. SOO, POS, PSS, and SOS were significantly different (*p* < 0.05) in cocoa butter and lard. The TAG profiles of fats resulted in a high proportion of unsaturated FAs for lard (55.06%) and cocoa butter (33.74%). Table 2 showed the TAG profiles when lard was added to cocoa butter from 1% to 30%. An adulterated sample with lard has additional TAGs viz. LLLn, LLL, OLL, OOL, PLL, OLL, POL, and POO. Cocoa butter adulterated with lard caused a slight increase in oleic-acid-predominating TAGs, while the palmitic acid containing TAGs decreased slightly. Kallio et al. [43] reported that lard has major saturated FA, especially palmitic acid, at the *sn-2* position, which made it distinct from other fats and oils.

### 3.2. Fatty Acid Methyl Ester Composition of Lard, Cocoa Butter, and Their Admixtures

Fatty acids (FAs) are essential components of edible fats and oils in which they can be found in the ester form with a glycerol backbone (triglycerides). Besides, FA compositions differ from one source to another. Therefore, FA profiles can be used for determining the purity or authenticity of animal fats. The quantitative analysis of FA composition is essential in food research with regards to the nutritional value content. In this study, the FAME compositions of lard, cocoa butter, and the mixture of lard in cocoa butter from 1% to 30% determined using GC-FID are presented in Table 3.

Three major fatty acids of lard (C18:0, C18:1, C18:2, and C16:0) in this study (0.36, 19.29, 32.41, 22.55) were lower compared to the values of 11.53, 24.64, and 17.29 reported by Nizar et al. [44]. However, the results in the present study concurred with those reported by Cheong et al. [45] and Nurjuliana et al. [46]. Edwards et al. [47] reported that fatty acid composition is influenced by the species, sex, and diet of animals.

Lard could be differentiated from cocoa butter having C10:0 (0.17%), C12:0 (1.44%), C15:0 (0.09%), C16:1 (1.22%), C17:0 (0.58%), and C18:3 (1.09%). Lard also has a higher total unsaturated fatty acid percentage (53.86%) compared to cocoa butter (33.74%). However, lard shared similar characteristics with that of cocoa butter having C16:0, C18:0, and C20:0, resulting in differences in their SFA contents. Cocoa butter has a total saturated fatty acid value of 66.27%.

Hence, from the above-mentioned findings, due to the addition of lard to cocoa butter from 1% to 30%, the amount of palmitic acid (C16:0), stearic acid (C18:0), capric acid (C10:0), lauric acid (C12:0), pentadecyclic acid (C15:0), palmitoleic acid (C16:1), margaric acid (C17:0), and linolenic acid (C18:3) increased. Increasing the proportion of lard in cocoa butter decreased the SFA from 65.66% to 59.23% and increased the USFA from 34.35% to 40.79%.

### 3.3. Thermal Analysis of Lard, Cocoa Butter, and Their Admixtures during Heating and Cooling Temperatures

**Figure 2:**
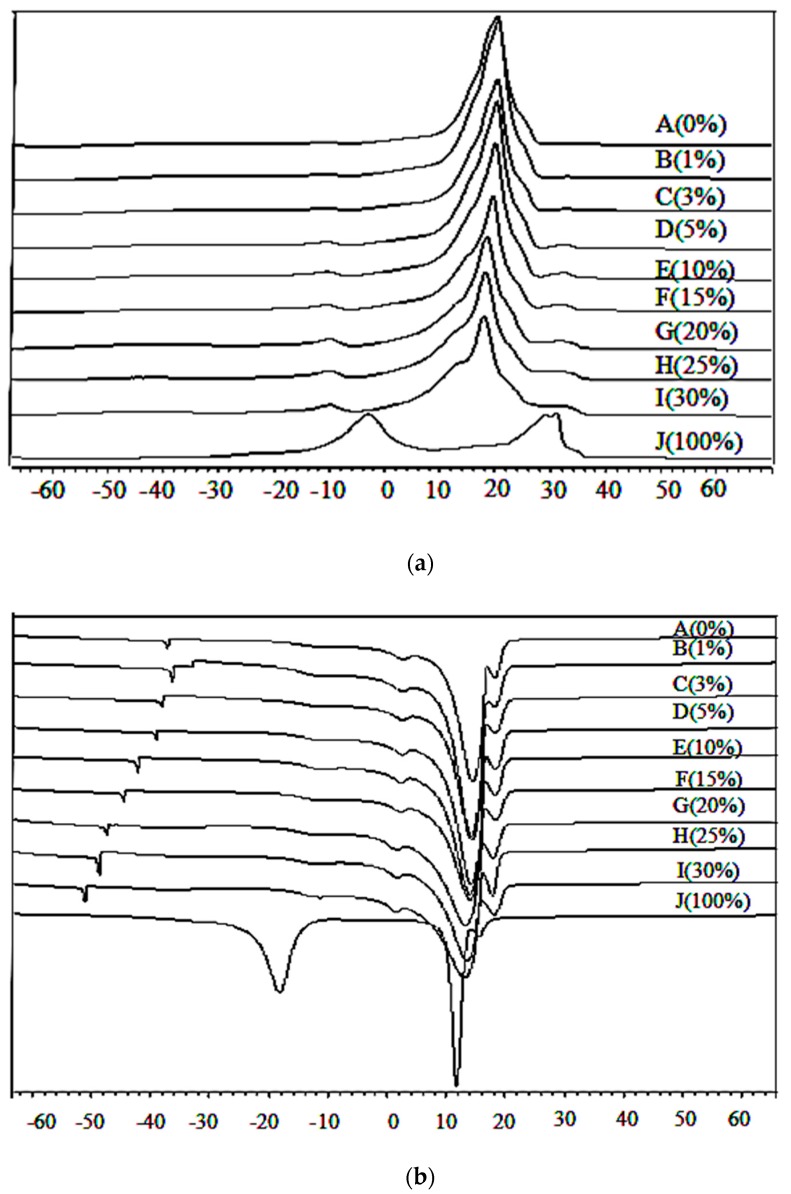
Differential scanning calorimetry (**a**) heating and (**b**) cooling thermogram of lard (LD; J: 100%), cocoa butter (CB; A: 0%), and their admixtures.

The DSC representative heating thermograms of lard, cocoa butter, and their admixtures in a range of 1% to 30% are as shown in Figure 2a. Lard and cocoa butter were totally different in their heating profiles. Lard (J) had two major peaks which appeared at −4.00 °C and 28.78 °C and a small shoulder peak at 34.09 °C. The heating thermal properties were acquired at the onset of the exothermal reaction, at the offset of the endothermal reaction, the peaks, and enthalpy. There is a significant difference (*p* < 0.05) between the heating enthalpy of lard (29.86 J/g) and cocoa butter (86.81 J/g). Cocoa butter (A) exhibited the highest T_off_ (23.38 °C) which could be due to a larger, highly saturated lipid fraction which melted at a higher temperature compared to those of more unsaturated lipids. Lard exhibited lower T_on_ (−10.30 °C) values, which indicated a higher amount of unsaturated fatty acid.

When cocoa butter is adulterated with lard, new groups of TAG with higher melting points could be introduced into the system, which may eventually change the original profile of cocoa butter. The heating curve of adulterant lard was also found to be completely different from the heating profile of cocoa butter. It was reported that any change in TAG composition influenced the thermal profiles of oils and fats [28]. Animal fats have different levels of saturated and unsaturated fatty acids, even though they have similar physical properties. Yanty et al. [48] reported that the more saturated the TAG, the higher the melting temperature.

There is one major endothermic peak at 20.16 °C and two minor endothermic peaks at −26.00 °C and 34.50 °C in cocoa butter adulterated with 3% to 30% lard influenced by the adulterant. As the concentration of lard increased from 3% to 30%, two minor peaks at −26.00 °C and 34.50 °C started to appear and a minor peak at 34.50 °C gradually overlapped with the neighbouring major peak. The major peak at 20.16 °C decreased in size resulting in a broader peak. Peak enthalpy tended to decrease from 84.53 J/g to 72.63 J/g as the adulterant increased from 1% to 30%. The heating profiles gave an indication of the amount of crystallized fat and the occurrence of polymorphic transitions [49]. The DSC heating profile has been used for determining the melting points and various polymorphic forms related to fat crystals. Compositional changes such as fatty acid chain length, the degree of unsaturation, and nature of the distribution of fatty acid in triacylglycerol species has an influence on phase transitions in fats and oils [22]. As reported by Che Man et al. [50], trisaturated TAG (SSS) melted at a higher temperature compared to triunsaturated TAG (UUU), monounsaturated (SSU), and diunsaturated (SUU) triglycerides.

The DSC cooling thermograms of lard, cocoa butter, and their mixture are shown in Figure 2b. The cooling profiles of lard displayed two marked exothermic peaks as reported by Chiavaro et al. [51]. Lard exhibited two major exothermic peaks observed at 17.99 °C and 11.98 °C, which are slightly higher compared to those reported by Nurrulhidayah et al. [26]. This difference could be due to the nature of lard, type of feeding, and variety of fatty acid composition. Cocoa butter exhibited one major exothermic peak at 14.58 °C and three small shoulder peaks appeared at −36.9 °C, 3.16 °C, and 17.97 °C. As reported by Dahimi et al. [52], this cooling behavior of pure samples could be attributed to the amount of saturated and unsaturated TAGs in the samples.

A summary of the cooling properties of lard, cocoa butter, and their mixture can be seen in Table 4. The onset of the cooling (T_on_) of lard is at −15.00 °C, while that of cocoa butter is at 16.94 °C. The cooling enthalpy for lard (−32.51 J/g) was significantly different (*p* < 0.05) from that of cocoa butter (−85.60 J/g). Lard has a lower cooling enthalpy (−32.51 J/g) due to the presence of free fatty acids and lipid oxidation products; these molecules will be absorbed into the crystal lattices of TAG, forming mixed crystals [53] which require lower enthalpy to undergo phase transition. The unsaturated FA and TAG will crystallize at a low temperature, while the saturated FA and TAG will crystallize at a higher temperature. The melting behavior of edible fats and oils varies due to the different characteristics of FA composition, as reported by Fasina et al. [54].

As lard adulteration increases from 1% to 30%, this shoulder peak at 17.97 °C gradually increases in size and shifts to a higher temperature. This shoulder peak is of particular interest due to its sensitivity to lard adulteration. In addition, cooling thermograms of adulterated cocoa butter with 1% to 30% lard showed a minor peak shift to a lower temperature from −36 °C to −41.5 °C. The major peak enthalpy decreased gradually from −85.41 J/g to a lower temperature (−78.67 J/g). Enlargement of the shoulder peaks of cocoa butter and lard which appeared at 17.97 °C could be due to the lower group melting of TAG. It was reported that the adulteration of cocoa butter with lard causes a shift in the peak temperature due to the binary mixture behaviour of the oil samples [21].

## 4. Conclusions

The results from this study revealed that increasing the lard concentration from 1% to 30% will increase the level of oleic acid (C18:1), while the amount of palmitic acid (C16:0) and stearic acid (C18:0) will decrease. With the addition of lard, the amount of POS, SOS, and POP decreased. An increased lard concentration from 1% to 30%, increased the total triunsaturated TAGs from 0.63% to 3.65%. A minor peak appeared around 34 °C with lard adulteration of 3% to 30%. Cooling thermograms of adulterated cocoa butter with 1% to 30% lard showed a minor peak shifted to a lower temperature of −36 °C to −41.5 °C. Increased lard concentration from 1% to 30% in cocoa butter increased the (C10:0), (C12:0), (C16:1), (C17:0), and (C18:3) fatty acids. The addition of lard increased the amount of LLLn, LLL, and OOL triglycerides. The thermal properties during heating were influenced by triglycerides and fatty acid compositions. Hence, the results from this study could be used as a basis for the identification of lard from other fats in the food authentication process.

## Figures and Tables

**Table 1:** Methods for the detection of lard in foods and food products.

Issues in Food Sample	Method of Detection	References
**DNA-based PCR method**		
Pork and lard in food products	cyt *b* PCR-RFLP	[4]
Lard in food products (sausages and casings, bread and biscuits)	cyt *b* PCR-RFLP	[5]
Lard detection in chocolate	Porcine-specific real-time PCR	[6]
**Fourier transform infrared spectroscopy**		
Lard mixed with other animal fats	FTIR with PLS	[7]
Lard mixed with animal fats	FTIR with PLS	[8]
Lard in cake formulation	FTIR with PLS	[9]
Lard in chocolate and chocolate products	FTIR with PLS	[2]
Lard in biscuit	FTIR with PLS	[10]
Lard mixed with lamb, cow and chicken body fats	FTIR with PLS and DA	[11]
Lard mixed with cod liver oil	FTIR with PLS and DA	[12]
Lard in other animal fats	FTIR with PLS and DA	[11]
Lard in virgin coconut oil (VCO)	FTIR with PLS and DA	[13]
Lard in vegetable oils	FTIR with PLS, PCR and DA	[14]
Lard in edible fats and oil	FTIR with PCA and CA	[15]
Lard in cream cosmetics	FTIR with PLS and PCR	[16]
Lard in frying oil	FTIR with PLS and DA	[17]
Lard in chocolate	FTIR with PLS and PCA	[18]
Lard in ink extracted from printed food packaging	FTIR with PCA and SIMCA	[19]
**Electronic nose technology**		
Lard in edible oil	E-nose	[20]
**Differential scanning calorimetry**		
Lard and randomized lard in RBD palm oil	DSC	[21]
Monitoring lard in canola oil	DSC	[22]
Lard adulteration	DSC	[22]
Lard in cooking oil	DSC	[23]
Lard in sunflower oil	DSC	[24]
Lard in canola oil	DSC	[25]
Lard in virgin coconut oil	DSC	[13]
Lard in butter	DSC	[26]
**Chromatographic-based techniques**		
Lard in meat products	HPLC	[27]
Lard in meat lipids	HPLC	[28]
Lard in animal fats and vegetable oils	HPLC	[28]
Lard in fried oils	HPLC	[29]
Lard in meat lipids	GLC (FID detector)	[30]
Lard in milk lipids	GLC (FID detector)	[31]
Lard in milk lipids	GLC (FID detector)	[32]
Lard in fried oils	GLC (FID detector)	[29]
Lard in animal fats	LC-MS	[33]
Lard in animal fats	GC-FID	[31]
Lard in vegetable oils	GC-FID	[28]
Lard in milk fat	GC	[34]
Lard in vegetable oils	GC (FID detector)	[28]
Lard in animal fats	GC×GC–TOF-MS	[35]
Lard in animal fats	GC×GC–TOF-MS	[36]

PCA: principal component analysis; DA: discriminant analysis; CA: cluster analysis; PCR: principle component regression; PLS: partial least square; SIMCA: soft independent modeling class analogy; RBD: refined, bleached, deodorized; PCR-RFLP: polymerase chain reaction-restriction fragment length polymorphism; GLC: gas liquid chromatography; FID: flame ionization detector; LC-MS: liquid chromatography–mass spectrometry; GC: gas chromatography; TOF: time-of-flight; MS: mass spectrometry.

**Table 2:** TAG composition of lard, cocoa butter, and their admixtures.

	TAGs	Lard Concentration (%)									
		0 (CB)	1	3	5	10	15	20	25	30	100 (LD)
	Unsaturated										
**Tri-unsaturated**	LLLn	nd < 0.04	0.04 (0.00) ^f^	0.05 (0.00) ^e,f^	0.05 (0.00) ^e,f^	0.06 (0.00) ^e^	0.07 (0.00) ^d^	0.11 (0.00) ^c^	0.35 (0.00) ^b^	0.36 (0.01) ^a^	0.88 (0.00) ^g^
	LLL	nd < 0.04	0.05 (0.00) ^h^	0.06 (0.00) ^g^	0.10 (0.00) ^f^	0.11 (0.00) ^e^	0.13 (0.00) ^d^	0.13 (0.00) ^c^	0.15 (0.00) ^b^	0.17 (0.00) ^a^	1.24 (0.01) ^h^
	OLL	nd < 0.04	0.05 (0.00) ^h^	0.08 (0.00) ^g^	0.15 (0.00) ^f^	0.25 (0.00) ^e^	0.35 (0.00) ^d^	0.51 (0.00) ^c^	0.52 (0.00) ^b^	0.85 (0.01) ^a^	2.94 (0.01) ^i^
	OOL	nd < 0.04	0.19 (0.00) ^h^	0.21 (0.01) ^g^	0.37 (0.00) ^f^	0.45 (0.00) ^e^	0.58 (0.00) ^d^	0.87 (0.00) ^c^	1.17 (0.00) ^b^	1.29 (0.00) ^a^	4.38 (0.01) ^i^
	OOO	0.24 (0.00) ^h^	0.30 (0.00) ^g^	0.32 (0.00) ^g^	0.35 (0.01) ^f^	0.42 (0.00) ^e^	0.58 (0.00) ^d^	0.73 (0.00) ^c^	0.93 (0.00) ^b^	0.98 (0.00) ^a^	2.28 (0.01) ^i^
	Sub total	0.24 (0.11)	0.63 (0.12)	0.72 (0.12)	1.02 (0.15)	1.29 (0.18)	1.71 (0.24)	2.35 (0.34)	3.12 (0.42)	3.65 (0.46)	11.72 (1.40)
**Di-unsaturated**	PLL	0.22 (0.01) ^g^	0.10 (0.00) ^i^	0.16 (0.00) ^h^	0.35 (0.00) ^f^	0.69 (0.00) ^e^	1.01 (0.00) ^d^	1.68 (0.01) ^c^	2.19 (0.01) ^b^	2.57 (0.02) ^a^	7.36 (0.08) ^j^
	POL	0.66 (0.00) ^i^	1.28 (0.00) ^h^	1.49 (0.00) ^g^	1.69 (0.00) ^f^	2.39 (0.01) ^e^	4.00 (0.00) ^d^	4.29 (0.01) ^c^	5.68 (0.01) ^b^	6.18 (0.00) ^a^	20.21 (0.01) ^j^
	POO	3.12 (0.01) ^i^	3.30 (0.00) ^h^	3.43 (0.01) ^g^	3.65 (0.01) ^f^	4.08 (0.01) ^e^	4.76 (0.01) ^d^	5.75 (0.01) ^c^	6.98 (0.01) ^b^	7.13 (0.03) ^a^	17.25 (0.01) ^j^
	SOO	3.46 (0.05) ^a^	3.40 (0.00) ^a^	3.32 (0.00) ^b^	3.21 (0.01) ^c^	3.19 (0.01) ^c^	3.14 (0.00) ^c,d^	3.11 (0.00)^d,e^	3.09 (0.01)^d,e^	3.05 (0.03) ^e^	3.21 (0.00) ^c^
	Sub total	7.46 (1.66)	8.08 (1.61)	8.40 (1.57)	8.90 (1.51)	10.35 (1.44)	12.91 (1.62)	14.83 (1.73)	17.94 (2.23)	18.93 (2.26)	48.03 (8.04)
	**Total unsaturated**	**7.70 (5.11)**	**8.71 (5.27)**	**9.12 (5.43)**	**9.92 (5.57)**	**11.64 (6.41)**	**14.62 (7.92)**	**17.18 (8.82)**	**21.06 (10.48)**	**22.58 (10.80)**	**59.75 (25.68)**
	**Saturated**										
**Di-saturated**	POP	19.13 (0.02) ^a^	18.77 (0.01) ^b^	18.64 (0.00) ^c^	18.56 (0.01) ^d^	18.47 (0.01) ^e^	18.31 (0.01) ^f^	18.01 (0.00) ^g^	17.64 (0.01) ^h^	17.29 (0.01) ^i^	3.21 (0.00) ^j^
	PPL	1.28 (0.00) ^g^	1.76 (0.00) ^f^	1.80 (0.01) ^f^	1.81 (0.01) ^f^	1.93 (0.02) ^e^	2.02 (0.00) ^d^	2.13 (0.04) ^c^	2.30 (0.00) ^b^	2.39 (0.01) ^a^	4.35 (0.00) ^h^
	POS	41.67 (0.01) ^a^	41.21 (0.01) ^b^	40.90 (0.01) ^c^	40.32 (0.01) ^d^	39.41 (0.01) ^e^	37.79 (0.01) ^f^	36.57 (0.04) ^g^	34.91 (0.01) ^h^	34.01 (0.01) ^i^	18.58 (0.01) ^j^
	SOS	28.47 (0.02) ^a^	27.67 (0.01) ^b^	27.55 (0.01) ^c^	27.37 (0.01) ^d^	26.51 (0.00) ^e^	25.15 (0.01) ^f^	23.87 (0.01) ^g^	21.71 (0.02) ^h^	20.96 (0.01) ^i^	1.32 (0.00) ^j^
	Sub total	90.55 (16.98)	89.41 (16.54)	88.89 (16.40)	88.06 (16.17)	86.32 (15.68)	83.27 (14.90)	80.58 (14.29)	76.56 (13.43)	74.65 (13.01)	27.46 (7.91)
**Tri-saturated**	PPS	0.27 (0.00) ^i^	0.32 (0.00) ^h^	0.40 (0.00) ^g^	0.46 (0.00) ^f^	0.51 (0.00) ^e^	0.58 (0.00) ^d^	0.68 (0.00) ^c^	0.74 (0.00) ^b^	1.09 (0.00) ^a^	1.99 (0.00) ^j^
	SSS	0.31 (0.00) ^i^	0.41 (0.01) ^h^	0.48 (0.00) ^g^	0.50 (0.00) ^f^	0.52 (0.00) ^e^	0.59 (0.00) ^d^	0.67 (0.00) ^c^	0.87 (0.00) ^b^	0.99 (0.00) ^a^	2.89 (0.01) ^j^
	PSS	1.17 (0.01) ^a^	1.15 (0.00) ^b^	1.11 (0.01) ^c^	1.06 (0.00) ^d^	1.01 (0.00) ^e^	0.93 (0.00) ^f^	0.90 (0.00) ^g^	0.79 (0.00) ^h^	0.69 (0.00) ^i^	nd <0.04
	Sub total	1.75 (0.51)	1.88 (0.46)	1.99 (0.39)	2.02 (0.34)	2.04 (0.29)	2.10 (0.20)	2.25 (0.13)	2.40 (0.07)	2.77 (0.21)	4.88 (1.46)
	**Total saturated**	**92.30 (62.79)**	**91.29 (61.89)**	**90.88 (61.45)**	**90.08 (60.84)**	**88.36 (59.59)**	**88.36 (59.59)**	**82.83 (55.39)**	**78.96 (52.44)**	**77.42 (50.83)**	**32.34 (15.97)**

Each value represents the mean ± SD of triplicate analyses; Means within the same row with different superscripts are significantly different (*p* < 0.05); Abbreviations: TAG, triacylglycerol; CB, cocoa butter; LD, lard; P, palmitic; O, oleic; L, linoleic; S, stearic; nd, not detected.

**Table 3:** Composition of fatty acids in lard, cocoa butter, and their admixtures.

FAs	Lard Concentration (%)									
	0 (CB)	1	3	5	10	15	20	25	30	100 (LD)
C10:0	0	0	0	0	0.01 (0.01) ^c^	0.02 (0.03) ^b,c^	0.06 (0.00) ^a,b^	0.06 (0.00) ^a,b^	0.08 (0.01) ^a^	0.17 (0.00) ^d^
C12:0	0	0.12 (0.00) ^e^	0.14 (0.01) ^d,e^	0.16 (0.00) ^d,e^	0.21 (0.00) ^c,d^	0.28 (0.01) ^c^	0.36 (0.06) ^b^	0.40 (0.00) ^a,b^	0.47 (0.01) ^a^	1.44 (0.00) ^f^
C15:0	0	0	0	0	0	0	0	0	0	0.09 (0.00) ^b^
C16:0	27.27 (0.07) ^a^	26.63 (0.08) ^b^	26.40 (0.00) ^c^	26.32 (0.02) ^c^	26.11 (0.07) ^d^	25.98 (0.04) ^d^	25.55 (0.03) ^e^	25.43 (0.02) ^e^	24.75 (0.04) ^f^	22.55 (0.00) ^g^
C16:1	0	0.25 (0.00) ^e^	0.26 (0.00) ^e^	0.28 (0.01) ^d,e^	0.30 (0.00) ^d^	0.36 (0.02) ^c^	0.40 (0.01) ^b^	0.42 (0.01) ^a,b^	0.44 (0.01) ^a^	1.22 (0.00) ^f^
C17:0	0	0.24 (0.00) ^e^	0.25 (0.01) ^e^	0.25 (0.00) ^d,e^	0.26 (0.00) ^d^	0.26 (0.00) ^d^	0.29 (0.00) ^c^	0.31 (0.01) ^b^	0.32 (0.00) ^a^	0.58 (0.00) ^f^
C18:0	37.75 (0.05) ^a^	37.45 (0.28) ^a,b^	37.09 (0.01) ^b,c^	36.74 (0.01) ^c^	36.03 (0.01) ^d^	35.24 (0.13) ^e^	34.10 (0.04) ^f^	33.66 (0.01) ^g^	32.75 (0.00) ^h^	0.36 (0.00) ^i^
C18:1	30.92 (0.06) ^e^	31.03 (0.21) ^e^	31.37 (0.00) ^d^	31.51 (0.00) ^c,d^	31.73 (0.07) ^b,c^	31.86 (0.00) ^a,b^	31.87 (0.01) ^a,b^	31.92 (0.02) ^a,b^	32.13 (0.01) ^a^	19.29 (0.09) ^f^
C18:2	2.82 (0.03) ^h^	2.88 (0.00) ^h^	3.10 (0.00) ^g^	3.36 (0.00) ^f^	3.96 (0.00) ^e^	4.67 (0.03) ^d^	6.04 (0.04) ^c^	6.48 (0.00) ^b^	7.83 (0.00) ^a^	32.41 (0.08) ^i^
C18:3	0	0.19 (0.00) ^e^	0.20 (0.00) ^d,e^	0.20 (0.00) ^d,e^	0.24 (0.00) ^c,d^	0.26 (0.01) ^c^	0.32 (0.03) ^b^	0.35 (0.00) ^a,b^	0.39 (0.01) ^a^	1.09 (0.00) ^f^
C20:0	1.25 (0.01) ^a^	1.22 (0.01) ^a,b^	1.21 (0.01) ^a,b^	1.19 (0.00) ^a,b^	1.16 (0.00) ^b^	1.09 (0.04) ^c^	1.03 (0.01) ^d^	1.00 (0.00) ^d^	0.86 (0.00) ^e^	0.82 (0.00) ^e^
**Total SFA**	66.27 (18.79)	65.66 (17.68)	65.09 (17.51)	64.66 (17.38)	63.78 (16.15)	62.87 (15.87)	61.39 (15.42)	60.86 (15.26)	59.23 (14.84)	44.94 (9.96)
**Total USFA**	33.74 (19.87)	34.35 (15.01)	34.93 (15.15)	35.35 (15.19)	36.23 (15.21)	37.15 (15.19)	38.63 (15.05)	39.17 (15.03)	40.79 (15.03)	53.86 (15.23)

Data are presented as means ± SD from triplicate determination. Means within the same row with different superscripts are significantly different (*p* < 0.05). Abbreviations: FAs, fatty acids; CB, cocoa butter; LD, lard; SFA, saturated fatty acids ; USFA, unsaturated fatty acids.

**Table 4:** Cooling and heating thermograms of cocoa butter, lard, and their admixtures.

Adulterated Samples (%)	Cooling Properties				Heating Properties			
	Onset (°C)	Offset (°C)	Enthalphy (J/g)	Peak (°C)	Onset (°C)	Offset (°C)	Enthalphy (J/g)	Peak (°C)
0 (CB)	16.94	9.49	−85.60	14.58	14.19	23.38	86.81	20.16
1	16.83	9.37	−85.41	14.41	13.78	23.36	84.53	20.11
3	16.71	9.18	−84.53	14.38	13.60	23.34	78.87	20.09
5	16.64	9.09	−83.55	14.27	13.53	23.21	77.04	20.06
10	16.55	8.47	−82.98	14.08	13.50	23.01	76.41	19.83
15	16.42	7.77	−82.31	14.05	13.48	22.33	75.01	19.43
20	16.26	6.65	−80.64	13.62	13.45	22.26	74.65	18.85
25	15.97	5.73	−79.70	13.30	13.40	21.95	74.15	18.10
30	15.70	5.08	−78.67	13.12	13.37	21.64	72.63	17.78
100 (LD)	−15.00	−22.16	−32.51	11.98	−10.30	2.38	29.86	−4.00

Abbreviations: CB, cocoa butter; LD, lard.

## References

[1] Food and Agriculture Organization (FAO) (2014). Assuring Food Safety and Quality.

[2] Che Man Y.B., Syaharizaa Z.A., Mirghania M.E.S., Jinapb S., Bakara J. (2005). Analysis of potential lard adulteration in chocolate and chocolate products using Fourier transform infrared spectroscopy. Food Chem..

[3] Kamal M., Karoui R. (2015). Analytical methods coupled with chemometric tools for determining the authenticity and detecting the adulteration of dairy products: A review. Trends Food Sci. Technol..

[4] Aida A.A., Che Man Y.B., Wong C.M.V.L., Raha A.R., Son R. (2005). Analysis of raw meats and fats of pigs using polymerase chain reaction for Halal authentication. Meat Sci..

[5] Aida A.A., Che Man Y.B., Raha A.R., Son R. (2007). Detection of pig derivatives in food products for Halal authentication by polymerase chain reaction—Restriction fragment length polymorphism. J. Sci. Food Agric..

[6] Rosman N., Mokhtar N.F.K., Ali M.E., Mustafa S. (2016). Inhibitory effect of chocolate components toward lard detection in chocolate using real time PCR. Int. J. Food Prop..

[7] Che Man Y., Mirghani M. (2001). Detection of lard mixed with body fats of chicken,lamb, and cow by fourier transform infrared spectroscopy. J.Am. Oil Chem. Soc..

[8] Jaswir I., Mirghani M.E.S., Hassan T.H., Mohd Said M.Z. (2003). Determination of lard in mixtures of body fats of mutton and cow by Fourier transform-infra red (FTIR) spectroscopy. J. Oleo Sci..

[9] Syahariza Z.A., Che Man Y.B., Selamat J., Bakar J. (2005). Detection of lard adulteration in cake formulation by Fourier transform infrared (FTIR) spectroscopy. Food Chem..

[10] Syahariza Z.A. (2006). Detection of Lard in Selected Food Model Systems Using Fourier Transform Infrared (FTIR) Spectroscopy. Master’s Thesis.

[11] Rohman A., Che Man Y.B. (2010). FTIR spectroscopy combined with chemometrics for analysis of lard in the mixtures with body fats of lamb, cow, and chicken. Int. Food Res. J..

[12] Rohman A., Che Man Y.B. (2009). Analysis of cod-liver oil adulteration using fourier transform infrared (FTIR) spectroscopy. J. Am. Oil Chem. Soc..

[13] Mansor T.S.T., Che Man Y.B., Rohman A. (2011). Application of fast chromatography and Fourier transform infrared spectroscopy for analysis of lard adulteration in virgin coconut oil. Food Anal. Methods.

[14] Rohman A., Che Man Y.B. (2011). Authentication analysis of cod liver oil from beef fat using fatty acid composition and FTIR spectra. Food Addit. Contam. Part A.

[15] Che Man Y.B., Rohman A. (2011). Differentiation of lard from other edible fats and oils by means of fourier transform infrared spectroscopy and chemometrics. J. Am. Oil Chem. Soc..

[16] Rohman A., Gupitasari I., Purwanto P., Triyana K., Rosman A.S., Ahmad S.A.S., Yusof F.M. (2014). Quantification of lard in the mixture with olive oil in cream cosmetics based on FTIR spectra and chemometrics. J. Teknol..

[17] Che Man Y.B., Marina A.M., Rohman A., Al-Kahtani H.A., Norazura O. (2014). A FTIR spectroscopy method for analysis of palm oil adulterated with lard in prefried French fries. Int. J. Food Prop..

[18] Suparman S., Rahayu W.S., Sundhani E., Saputri S.D. (2015). The use of Fourier transform infrared spectroscopy (FTIR) and gas chromatography mass spectroscopy (GCMS) for Halal authentication in imported chocolate with various variants. J. Food Pharm. Sci..

[19] Ramli S., Talib R.A., Rahman R.A., Zainuddin N., Othman S.H., Rashid N.M. (2015). Detection of lard in ink extracted from printed food packaging using fourier transform infrared spectroscopy and multivariate analysis. J. Spectrosc..

[20] Che Man Y.B., Gan H.L., Nor Aini I., Nazimah S.A.H., Tan C.P. (2005). Detection of lard adulteration in RBD palm olein using an electronic nose. Food Chem..

[21] Marikkar J.M.N., Lai O.M., Ghazali H.M., CheMan Y.B. (2001). Detection of lard and randomized lard as adulterants in refined-bleached-deodorized palm oil by differential scanning calorimetry. J. Am. Oil Chem. Soc..

[22] Marikkar J.M.N., Ghazali H.M., Che Man Y.B., Lai O.M. (2002). The use of cooling and heating thermograms for monitoring of tallow, lard and chicken fat adulterations in canola oil. Food Res. Int..

[23] Mansor T.S.T., Che Man Y.B., Shuhaimi M. (2012). Employment of differential scanning calorimetry in detecting lard adulteration in virgin coconut oil. J. Am. Oil Chem.Soc..

[24] Marikkar J.M.N., Dzulkifly M.H., Nadiha M.Z.N., Man Y.B.C. (2012). Detection of animal fat contaminations in sunflower oil by differential scanning calorimetry. Int. J. Food Prop..

[25] Marikkar J.M.N., Rana S. (2014). Use of differential scanning calorimetry to detect canola oil (*Brassica napus* L.) adulterated with lard stearin. J. Oleo Sci..

[26] Nurrulhidayah A.F., Arieff S.R., Rohman A., Amin I., Shuhaimi M., Khatib A. (2015). Detection of butter adulteration with lard using differential scanning calorimetry. Int. Food Res. J..

[27] Rashood K.A., Shaaban R.R.A., Moety E.M.A., Rauf A. (1996). Compositional and thermal characterization of genuine and randomized lard: A comparative study. J. Am. Oil Chem. Soc..

[28] Marikkar J.M.N., Ghazali H.M., Che Man Y.B., Peiris T.S.G., Lai O.M. (2005). Distinguishing lard from other animal fats in admixtures of some vegetable oils using liquid chromatographic data coupled with multivariate data analysis. Food Chem..

[29] Marikkar J.M.N., Ghazali H.M., Long K., Lai O.M. (2003). Lard uptake and its detection in selected food products deep-fried in lard. Food Res. Int..

[30] Saeed T., Ali S.C., Rahman H.A., Saway W.N. (1989). Detection of pork and lard as adulterants in processed meat: Liquid chromatographic analysis of derivatized triglycerides. J. Assoc. Off. Anal. Chem..

[31] Farag R.S., Abo-raya S.H., Ahmed F.A., Hewedi F.M., Khalifa H.H. (1983). Fractional crystallization and gas chromatographic analysis of fatty acids as a means of detecting butterfat adulteration. J. Am. Oil Chem. Soc..

[32] Farag R.S., Ahmed F.A., Shihata J.A.A., Abo­raya S.H., Abdalla A.F. (1982). Use of unsaponifiable matter for detection of ghee adulteration with other fats. J. Am. Oil Chem. Soc..

[33] Dugo P., Kumm T., Fazio A., Dugo G., Mondello L. (2006). Determination of beef tallow in lard through a multidimensional off-line non-aqueous reversed phase-argentation LC method coupled to mass spectrometry. J. Sep. Sci..

[34] Goudjil H., Fontecha J., Fraga M.J., Juarez M. (2003). TAG composition of ewe’s milk fat, Detection of foreign fats. J. Am.Oil Chem. Soc..

[35] Indrasti D., Che Man Y.B., Mustafa S., Hashim D.M. (2010). Lard detection based on fatty acids profile using comprehensive gas chromatography hyphenated with time-of-flight mass spectrometry. Food Chem..

[36] Chin S.T., Che Man Y.B., Tan C.P., Hashim D.M. (2009). Rapid profiling of animal-derived fatty acids using fast GC 3 GC coupled to time-of-flight mass spectrometry. J. Am. Oil Chem. Soc..

[37] Hoffmann G. (1989). The Chemistry and Technology of Edible Oils and Fats and Their High Fat Products.

[38] Sionek B., Krygier K., Ukalski K., Ukalska J., Amarowicz R. (2013). The influence of nitrogen and carbon dioxide on the oxidative stability of fully refined rapeseed oil. Eur. J. Lipid Sci. Technol..

[39] Haryati T., Che Man Y.B., Ghazali H.M., Asbi B.A., Buana L. (1998). Determination of iodine value of palm oil based on triglyceride composition. J. Am. Oil Chem. Soc..

[40] Marina A.M., Che Man Y.B., Nazimah S.A.H., Amin I. (2009). Monitoring adulteration of virgin coconut oil by selected vegetable oils using differential scanning calorimetry. J. Food Lipids.

[41] Tan C.P., Che Man Y.B. (2002). Comparative differential scanning calorimetric analysis of vegetable oils: I. Effects of heating rate variation. Phytochem. Anal..

[42] Shukla V.K.L. (1995). Cocoa butter properties and quality. Lipid Technol..

[43] Kallio H., Yli-Jokipli K., Kurvinen J.P., Sjovall O., Tahvonen R. (2001). Regioisomerism of triacylglycerols in lard, tallow, yolk, chicken skin, palm oil, palm olein, palm stearin, and transesterified blend of palm stearin and coconut oil analyzed by tandem mass spectrometry. J. Agric. Food Chem..

[44] Nizar N.N.A., Marikkar J.M.N., Hashim D.M. (2013). Differentiation of lard, chicken fat, beef fat and mutton fat by GCMS and EA-IRMS techniques. J. Oleo Sci..

[45] Cheong L.Z., Hong Z., Lise N., Jensen K., Haagensen J.A., Xuebing X. (2010). Physical and sensory characteristics of pork sausages from enzymatically modified blends of lard and rapeseed oil during storage. Meat Sci..

[46] Nurjuliana M., Che Man Y.B., Hashim D.M. (2011). Analysis of lard’s aroma by an electronic nose for rapid halal authentication. J. Am. Oil Chem. Soc..

[47] Edwards H.M.J., Denman F. (1975). Carcass composition studies. 2. Influence of breed, sex and diet on gross composition of the carcass and fatty acid composition of adipose tissue. Poult. Sci..

[48] Yanty N.A.M., Marikkar J.M.N., Che Man Y.B., Long K. (2011). Composition and thermal analysis of lard stearin and lard olein. J. Oleo Sci..

[49] Fredrick E., Foubert I., Van De Sype J., Dewettinck K. (2008). Influence of monoglycerides on the crystallization behavior of palm oil. Cryst. Growth Des..

[50] Che Man Y.B., Hariyati T., Ghazali H.M., Asbi B.A. (1999). Compositional and thermal profile of crude palm oil and its products. J. Am. Oil Chem. Soc..

[51] Chiavaro E., Vettadini E., Estrada M.T.R., Cerretani L., Bendini A. (2008). Differential scanning calorimeter application to the detectionof refined hazelnut oil in extra virgin olive oil. Food Chem..

[52] Dahimi O., Rahim A.A., Abdulkarim S.M., Hassan M.S., Hashari S.B., Mashitoh A.S. (2014). Multivariate statistical analysis treatment of DSC thermal properties for animal fat adulteration. Food Chem..

[53] Jacobsberg B., Ho O.C. (1976). Studies in palm crystallization. J. Am. Oil Chem. Soc..

[54] Fasina O.O., Craig-Schmidt M., Colley Z., Hallman H. (2008). Predicting melting characteristics of vegetable oils from fatty acid composition. LWT Food Sci. Technol..

